# Selenium and Its Supplementation in Cardiovascular Disease—What do We Know?

**DOI:** 10.3390/nu7053094

**Published:** 2015-04-27

**Authors:** Carina Benstoem, Andreas Goetzenich, Sandra Kraemer, Sebastian Borosch, William Manzanares, Gil Hardy, Christian Stoppe

**Affiliations:** 1 Department of Thoracic and Cardiovascular Surgery, University Hospital RWTH Aachen, Pauwelsstrasse 30, 52074 Aachen, Germany, E-Mails: agoetzenich@ukaachen.de (A.G.), skraemer@ukaachen.de (S.K.); sborosch@ukaachen.de (S.B.); 2 Department of Critical Care, Intensive Care Unit, Faculty of Medicine, Universidad de la República (UdeLaR), Italia Av. 14th floor. 11.600, Montevideo, Uruguay; E-Mail: wmanzanares@adinet.com.uy; 3 Clinical Nutrition, College of Health, Massey University, Albany Campus Private Bag 102 904 Auckland 0632, New Zealand; E-Mail: g.hardy@massey.ac.nz; 4 Institute of Biochemistry and Molecular Cell Biology, RWTH Aachen University, 52074 Aachen, Germany; 5 Department of Anesthesiology, University Hospital RWTH Aachen, Pauwelsstrasse 30, 52074 Aachen, Germany

**Keywords:** cardiovascular disease, coronary heart disease, cardiac surgery, selenium selenoproteins

## Abstract

The trace element selenium is of high importance for many of the body’s regulatory and metabolic functions. Balanced selenium levels are essential, whereas dysregulation can cause harm. A rapidly increasing number of studies characterizes the wide range of selenium dependent functions in the human body and elucidates the complex and multiple physiological and pathophysiological interactions of selenium and selenoproteins. For the majority of selenium dependent enzymes, several biological functions have already been identified, like regulation of the inflammatory response, antioxidant properties and the proliferation/differentiation of immune cells. Although the potential role of selenium in the development and progression of cardiovascular disease has been investigated for decades, both observational and interventional studies of selenium supplementation remain inconclusive and are considered in this review. This review covers current knowledge of the role of selenium and selenoproteins in the human body and its functional role in the cardiovascular system. The relationships between selenium intake/status and various health outcomes, in particular cardiomyopathy, myocardial ischemia/infarction and reperfusion injury are reviewed. We describe, in depth, selenium as a biomarker in coronary heart disease and highlight the significance of selenium supplementation for patients undergoing cardiac surgery.

## 1. Introduction

The essential trace element selenium (Se) is crucial for many biological functions including thyroid hormone metabolism, the body’s antioxidant defense systems, the adaptive and acquired immune system and prevention of certain cancers. Accumulating evidence suggests that selenium is also of importance for optimal functioning of the cardiovascular system. First hints of its central role date back to the 1960s when its functional role in the development of white muscle disease was revealed [1]. Selenium is therefore today regarded as an essential component of many dietary supplements, although its role in cardiovascular disease remains only partly understood.

Balanced selenium levels are needed for various biological functions in the human body, but very low or very high levels of selenium intake can cause deleterious effects. Intake depends locally on the selenium content of the soil, on which crops, which represent an essential part of daily food, grow [2]. Consequently, worldwide regions have been characterized by different selenium soil levels that correspond with whole blood selenium levels and selenium content of hair samples taken from healthy volunteers [3,4]. A chronic selenium deficiency was discovered first about 80 years ago in a province of north-western China [5]. Patients were suffering from a rapidly progressive cardiomyopathy, resulting in extensive fibrosis and degenerative changes, today known as Keshan disease after the Chinese region [6]. In contrast to selenium deficiency, selenium toxicity is observed less commonly and results from accidental/suicidal ingestion or in most cases from a chronic over-supplementation, due to high selenium content in the daily food [6]. Patients with selenosis present with brittle nails and hair as well as garlic smell in the exhaled air [6,7]. Other symptoms of an acute selenium overdose are rather unspecific like vomiting, dizziness and pulmonary edema [8].

Selenium mediates its effects mainly through incorporation into different selenoproteins. For the majority of selenium dependent enzymes, a wide range of biological functions have already been identified, like regulation of the inflammatory response and proliferation and differentiation of several immune cells [9,10,11]. Moreover, previous studies have demonstrated that selenium supplementation may affect and thus control the migration, adherence and phagocytosis of leucocytes [12]. Selenium is considered a cornerstone of the body’s antioxidant defense mechanism [13]. When incorporated into the various selenoenzymes, selenium increases antioxidant capacity and influences the inflammatory signaling pathways that modulate reactive oxygen species (ROS) by inhibiting the nuclear factor-kappa B (NF-κB) cascade, resulting in a suppressed production of interleukins and tumor necrosis factor alpha (TNF-α) [14]. The majority of selenoproteins are classified as antioxidants that regulate various signaling processes by influencing the redox homeostasis and cellular Ca^2+^ influx [15].

A rapidly increasing number of studies further characterize the wide range of selenium dependent functions in the human body and elucidate the complex and multiple physiological and pathophysiological interactions of selenium and/or its proteins. It still remains to be clarified, at a cellular and molecular level, how a significant selenium deficiency or variations in dietary selenium may affect these important biological functions, especially in the cardiovascular system. Although the potential role of selenium in the development and progression of cardiovascular diseases has been investigated, both observational studies and data from different selenium supplementation studies remain inconclusive and will be considered in this review [16,17]. The potential reasons for this discrepancy are multifactorial and may be due to the influence of other antioxidants, large variance in supplementation strategies and the different selenium formulas. Although several earlier reviews attempted to address these questions, few have considered any potential differences between selenium, applied in its active inorganic form as selenite, and organic derivatives, which need further *in vivo* modifications before exerting any biological functions [18]. Selenium content in foods and dietary supplements exists in different chemical forms (organic and inorganic selenocompounds) including selenomethionine (Se-Met), selenocysteine (Se-Cys), as well as selenite, selenious acid, and sodium selenite (mainly supplements). Bioavailability and pharmacokinetic profiles of selenium depend on the administered selenocompounds. In this regard, Se-Met is one of the most effective organic selenocompound for improving selenium status, as Se-Met is non-specifically incorporated into proteins. Nonetheless, Se-Met is a less-efficient metabolic source than inorganic forms of selenium, since it needs to be reduced, via Se-Cys, to hydrogen selenide (H_2_Se), which is considered a key precursor in the metabolic interconversions of both organic and inorganic selenocompounds [19]. Despite this limitation, organic selenocompounds are frequently preferred in short-term therapy as they are less acutely toxic. Moreover, Se-Met is not available for intravenous therapy [20]. To date, inorganic selenocompound supplements have proven to be the most efficacious parenteral forms of selenium supplementation for optimizing selenoenzymes activity in different animal and clinical studies [20]. Standardized formulations and protocols are still needed to enable a high-quality research comparison of selenium supplements and to determine the best form of selenium for cardiovascular diseases.

## 2. The Role of Selenium and Selenoproteins in the Human Body

So far, over 25 selenoproteins have been identified that play diverse roles in the regulation of cellular redox processes. They are expressed in a variety of tissues and cells and exhibit numerous functions [21]. Glutathione peroxidases (GPx) detoxify intracellular hydrogen peroxide thus protecting the cell from lipoprotein and/or DNA damage while thioredoxin reductases (TrxR) regenerate thioredoxin and thereby balance the redox status of the cell. One subfamiliy, including SelW, SelV, SelT and SelH, forms mixed disulfides with substrate proteins and bind DNA in a redox-sensitive manner. Selenoprotein T (SelT) has been suggested to be involved in calcium mobilization and glucose metabolism [22,23], whereas SelM and Sep15 function as oxidoreductases in the ER lumen [21]. Since the general function of selenoproteins has extensively been reviewed before [24,25], we will focus here on those selenoproteins involved in cardiovascular stress response.

### 2.1. Glutathione Peroxidases

**Figure 1:**
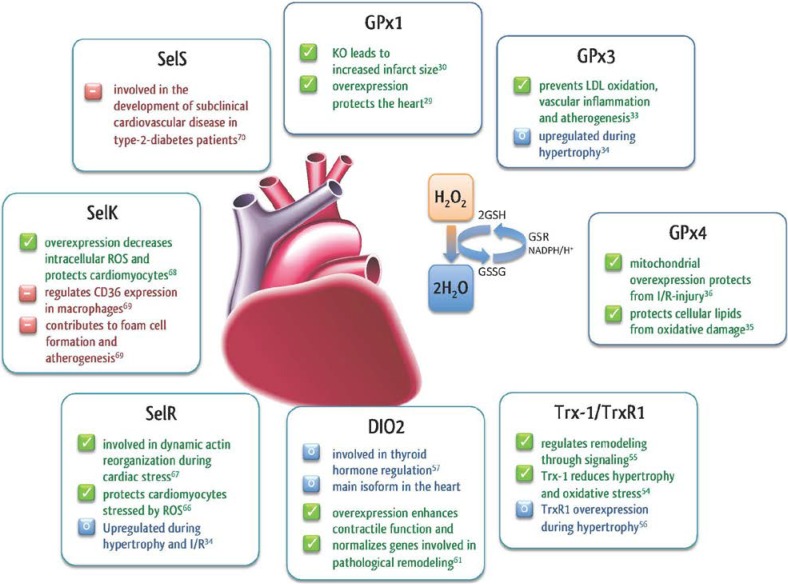
Roles of selenoproteins in the heart.

GPx are considered the most important proteins within the selenoprotein family. In contrast to other antioxidants, they can neutralize reactive oxygen and reactive nitrogen species (Figure 1).

To catalyze the reduction of H_2_O_2_ to water by glutathione peroxidases, reduced glutathione (GSH), is needed as co-factor. During detoxification, GSH is converted to the dimer, oxidized glutathione (GSSG), which in turn rejuvenates GSH, catalyzed by glutathione reductase and NADPH/H^+^ (Figure 1). In humans, the GPx family consists of five Sec-containing enzymes: GPx1 (cytosolic), GPx2 (intestinal), GPx3 (plasma), GPx4 (membrane), and GPx6 (olfactory) [26]. The first designated mammalian selenoprotein was GPx1 [27,28], which plays a critical role in prevention of ischemia/reperfusion (I/R) related injury and regulates the redox balance. In a transgenic mouse model, it was shown that GPx1 over expression results in a better protection from myocardial I/R injury [29]. The importance of GPx1 in I/R injury was further confirmed in an animal model. Genetic deletion of GPx1 results in a significantly reduced protection against I/R associated complications in GPx1^−/−^ mice. Furthermore, the size of infarcts in GPx1^−/−^ mice were larger when compared to those of normal wildtype mice [30]. More recent studies have indicated that female mice are less susceptible to I/R resulting damage than male mice [31].

Because of its presence in human plasma, GPx3 is widely used as a marker for selenium status. To study the influence of GPx3 Jin and co-workers [32] used a no-flow I/R stroke model. Importantly, the cerebral infarcts of GPx3^−/−^ mice were much larger than those of the control group. The authors suggested that the created strokes were platelet dependent. This study indicates the relevance of GPx3 in endothelial function and for perpetuating normal platelet inhibition [32]. Furthermore, it was shown that GPx3 inhibits the oxidation of plasma LDL by removing soluble hydroperoxides. GPx3 thereby prevents vascular inflammation and atherogenesis [33]. On the other hand, GPx3 was upregulated in a mouse model of cardiac hypertrophy [34].

The intracellular enzyme GPx4 is capable of reducing complex hydroperoxides in membrane bilayers [35]. By using a GPx4 overexpression model, Hollander and co-workers [36] propose that GPx4 protects against I/R associated injuries, mainly in mitochondria of neonatal rat cardiac myocytes [37].

### 2.2. Thioredoxin Reductase (TrxR)

Thioredoxins are enzymes that regulate numerous redox processes in the cell, including signaling, cell–cell communication, as well as DNA metabolism and repair [38,39]. Their antioxidant function is mediated by cysteine thiol-disulfide exchange, which leads to the reduction of proteins and maintains a reduced environment in the cell [40]. TrxR regenerate reduced thioredoxins in a NADPH/H^+^ dependent manner [41]. Therefore, the Trx-1/TrxR system is one of the most important mechanisms to control cellular redox balance. So far, three selenoenzymes of the TrxR family have been identified: TrxR1 which is located in the cytosol [42], TrxR2 located in the mitochondria [43] and TrxR3 [44], which is testis-specific. Additionally to thioredoxin, TrxRs react with a wide variety of substrates; including Trx fold proteins like protein disulfide-isomerase (PDI) and other PDI-family members [45], calcium-binding protein 1 and 2 (CaBP1 and CaBP2) [46] but also other selenium containing compounds, such as selenocystine [47], selenodiglutathione [48], methylselenate [49], selenite [50] and ebselen. Metabolism of most of these compounds results in production of hydrogen selenide, which is the selenium donor for Sec synthesis. Thus, the TrxR system is also involved in selenoprotein synthesis [51].

When the redox balance is shifted towards an oxidative state, hypertrophy and apoptotic signaling are activated in cardiomyocytes [52]. Thioredoxins, especially Trx-1/TrxR1 are known to regulate cardiac functions and are involved in cardiovascular diseases [53]. Trx-1 was shown to reduce hypertrophy as well as oxidative stress in response to pressure overload and the dominant negative protein increased oxidative stress and induced cardiac hypertrophy [54]. Induction of myocardial hypertrophy also induces TrxR1 expression whereas mitochondrial TrxR2 is constitutively expressed [34]. The Trx-1/TrxR1 system also plays a major role during cardiac remodeling by regulating signaling events like the S-thiolation of Ras [53,55,56]. In summary, this implies that selenium, in the form of Trx-1/TrxR1, may regulate myocardial remodeling by reversibly reducing signaling molecules.

### 2.3. Thyroid Hormone Deiodinases (DIO)

The iodothyronin deiodinases are an oxidoreductase family consisting of three Sec-containing isoforms (DIO1, 2 and 3) that are involved in thyroid hormone regulation [57]. The isoforms are expressed in a wide variety of adult and fetal tissues, with little expression in immune cells [58]. They catalyze the release of iodine directly from the thyronine hormones and contribute to activation and inactivation of the initially released hormone precursor T4 (thyroxine) and T3 (triiodothyronine). DIO1 and DIO2 initiate the thyroid hormone action by converting T4 to T3 whereas DIO3 inactivates T4 and T3 irreversibly [59]. However, the concentration of active thyroid hormone may affect the amount of selenium available for selenoprotein synthesis and thus, DIOs may play an indirect role in inflammation and immunity.

Since thyroid hormone metabolism is important for the development and health of the mature heart, dysregulation of thyroid hormone levels may result in cardiovascular perturbations like hypertrophy, increased heart rate and contractility [60]. DIO2 is the main DIO isoform in the heart and is important for cardiac function. Overexpression of cardiac DIO2 in a mouse model caused enhanced contractile function, preserved heart function and normalized the expression of several genes involved in pathological remodeling [61].

### 2.4. Selenoprotein R (SelR, MsrB1)

SelR, also known as methionine-sulfoxide reductase B1 (MsrB1), belongs, together with the non-selenoprotein members MsrA, B2 and B3, to the antioxidant Msr family that reduces oxidized methionine residues on proteins. ROS reversibly oxidizes methionine to methionine-sulfoxides and SelR reduces the methionine residues again. This reversible oxidation and reduction has been shown to be an important regulator of the activity of signaling molecules, thereby controlling redox processes in the cell. In addition, these cycles lead to consumption of ROS and thereby increases the resistance of proteins to oxidation [62]. In general it has been shown that a decrease of SelR activity leads to a decreased resistance to oxidative stress, whereas over expression increased resistance to oxidative stress [63,64].

SelR is expressed in a wide variety of tissues and cells, predominantly in the nucleus and cytoplasm and may be involved in the regulation of cardiac stress [65]. It is the only enzyme of the Msr family that is highly up regulated in murine models of cardiac hypertrophy and ischemia reperfusion suggesting an important regulatory role of SelR during cardiac stress [34,66]. ROS-induced formation of methionine sulfoxides may increase SelR expression and thus minimize the damage to stressed cardiomyocytes. SelR is also involved in the dynamic actin reorganization during cardiac stress [67].

### 2.5. Other Selenoproteins in the Heart

In addition to GPxs, TrxRs, and SelR, other selenoproteins are important during cardiac stress. SelK is a selenoprotein of the endoplasmatic reticulum (ER) membrane and overexpression was shown to decrease intracellular levels of ROS and protected cardiomyocytes from oxidative stress [68]. On the other hand, SelK seems to promote atherogenesis. Knockout of SelK in macrophages led to a reduction of cluster of differentiation 36 (CD36) expression and thereby promoted foam cell formation [69].

**Figure 2:**
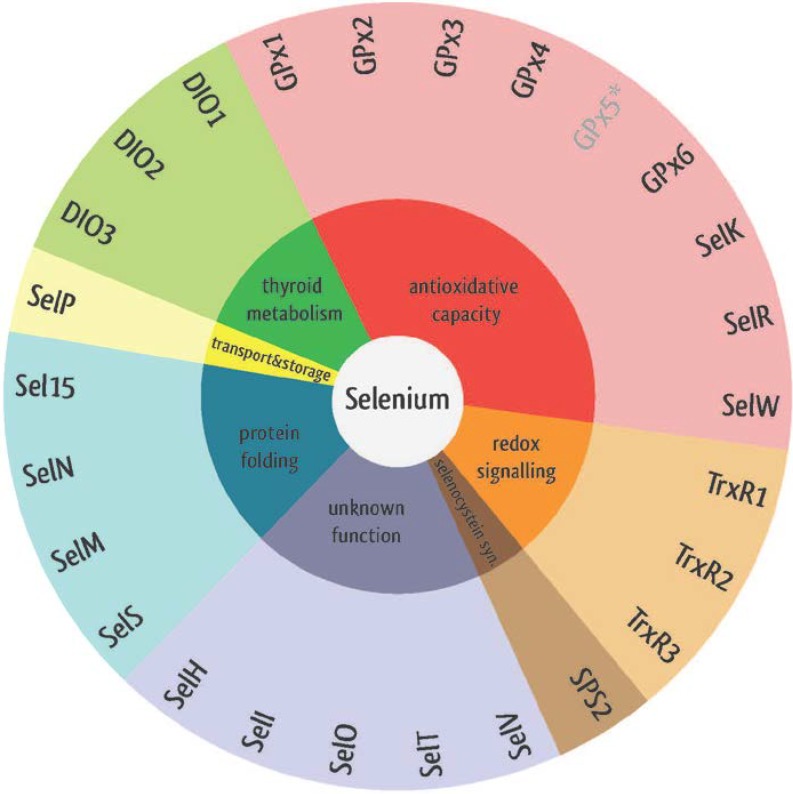
Classes of selenoproteins and their putative functions.

For another ER membrane selenoprotein named SelS, it has been suggested that a specific gene polymorphism plays a role in the development of subclinical cardiovascular disease during type-2 diabetes [70]. Both selenoproteins are related to inflammation and regulation of ER stress induced by misfolded proteins [71], which is an important factor regulating cardiomyocyte fate. Figure 2 provides an overview of all identified selenoproteins with their putative functions.

## 3. The Functional Role of Selenium in the Cardiovascular System

### 3.1. The Role of Selenium in the Development of Cardiomyopathy—Keshan Disease

The first hints about the significance of selenium in the cardiovascular system go back to earlier reports about a rapidly progressive and severe cardiomyopathy (Keshan Disease), which is characterized by myocardial necrosis and calcification [72]. Although selenium deficiency obviously appears to represent the primary pathogenic factor in the development and occurrence of this disease, it was subsequently considered more likely to be a conditional predisposing factor than an etiologic factor for this form of juvenile cardiomyopathy. In this context, previous studies demonstrated that selenium supplementation in deficient mice, reduced the cardiotoxicity of the coxsackie b virus that was previously isolated from patients with Keshan disease. Subsequent studies confirmed these findings and showed an increased susceptibility of mice to the development of viral-induced cardiomyopathy, when fed with a selenium-deficient diet [73,74,75].

Although the exact mechanisms remain vague, more recent studies have demonstrated the protective properties of GPx activity on the disease development and reported an increased sensitivity to this viral infection in mice with 50% of GPx1 knockout, whereas wildtype mice remained resistant [76].

### 3.2. The Role of Selenium and Selenoproteins in Myocardial I/R

In general, the GPx family belongs to the best-characterized selenoproteins in the context of cardiovascular biology. Experiments that focused on the importance of selenium deficiency in the development of cardiovascular diseases—without infectious origin—have indicated that the association between low selenium intake and cardiovascular pathologies might result from increased oxidative stress and its sequelae. Animal studies using different dosages and formulas of selenium, as well as studies in GPx knockout mice have revealed its crucial function in neutralization of reactive oxygen and nitrogen species, thereby limiting the organ injury after myocardial ischemia/reperfusion. Beside the GPx isoforms, thioredoxin reductase is thought to provide regulatory functions in the cardiovascular system through oxidation of intra- and extracellular signaling molecules [39] with impact on adaptive mechanisms such as remodeling [55].

The exact role of other selenoproteins on the cardiovascular system and disease development remains only partly understood. Lu and co-workers provided the first evidence that selenoprotein K contributes to the antioxidant defense mechanisms in cardiomyocytes [68]. In this connection, Venardos and colleagues demonstrated in a rat model that selenium deficiency leads to an increased myocardial injury with amplified protein and lipid peroxidation after myocardial I/R [77]. These data were further supported by the experiments of Tanguy and co-workers, which showed that a selenium deficiency in rats lead to an increased myocardial damage and altered recovery of the cardiac function after myocardial I/R. The underlying reasons for this observation were due to a decreased GPx activity measured in the blood and cardiomyocytes [78,79,80]. Given these findings, various experimental studies aimed to limit the known myocardial I/R injury through a selenium supplementation via a selenium-enriched diet. Indeed, the Venardos group showed significantly reduced myocardial I/R injury in rats compared to animals fed with a low selenium diet [81]. Furthermore, Tanguy and colleagues confirmed selenium’s protective characteristics and demonstrated an improved cardiac functional recovery, significantly reduced infarct size and decreased incidence of post-ischemic ventricular arrhythmias in rats that received the highest selenium intake [82,83,84,85]. The underlying reasons for these observations remain only partly understood. Since I/R related oxidative stress is recognized as a major factor contributing to the alteration in recovery, increased infarct size and pathogenesis of post-ischemic arrhythmias, it is supposed that selenium provides its protective properties mainly as an essential co-factor of many antioxidants. However, additional research is needed to further investigate the selenium dependent effects at a molecular level.

### 3.3. The Role of Selenium in Cardiovascular Disease

Selenium plays an essential part in the selenoprotein-induced defense system. Consequently, selenium blood levels have been widely utilized as a biomarker for oxidative stress-associated diseases. Various observational studies have investigated the significance of serum selenium levels on the development of cardiovascular diseases.

#### 3.3.1. Selenium as Biomarker

Oxidative stress plays a pivotal role in the chronic as well as the acute phase of coronary heart disease (CHD). Serum levels of selenium are known to be positively correlated with the activity of GPx [28,86] and other antioxidant selenoproteins, which are crucial for maintenance of redox homeostasis and optimal antioxidant defense. In the chronic development of CHD, reduced selenium levels thus may result in an inadequate prevention of LDL oxidation, (which is the major cause for the development of atherosclerotic plaques), through uptake by endothelial cells and macrophages [87]. Since adequate selenium levels depend on the daily food intake, it was observed that the occurrence of heart disease was associated with low selenium levels, which result in a suboptimal activity of GPx. In 2006, a meta-analysis by Flores-Mateo *et al.* concluded that there was an inverse association between selenium concentrations and the incidence of CHD. However, the analyzed observational studies were of uncertain validity [16]. Another meta-analysis of 13 prospective cohort studies found a moderate inverse relationship between plasma/serum selenium and CHD but pointed out a limited interpretability due to potential biases in the protocols [88]. In contrast, Xun and co-workers found no associations between toenail selenium levels and measures of sub-clinical atherosclerosis among young American adults [89]. An interesting Finnish report evaluated CHD data from the nationwide supplementation of selenium-enriched fertilizers in Finland since the 1980s: Although serum selenium levels were raised to a level considered to be optimal (1.40 μmol L^−1^), rates of cardiovascular disease remained similar during the pre- and post-supplementation periods [90]. To account for these conflicting observational reports plus the long term follow up data from the US National Health and Nutrition Examination Survey (the NHANES study), a U-shaped relationship between serum selenium levels and cardiovascular mortality was proposed [91,92].

With increasing evidence for the role of oxidative stress in the pathophysiology of hypertension, antioxidants have been suggested as a possible co-treatment for CHD [93]. However, evidence showing any association between blood selenium and arterial blood pressure in humans are contradictory. On the one hand, elevated baseline selenium levels were found to be associated with a lower risk for hypertension in men [94]. On the other hand, data from the Lipid Analytic Cologne cohort suggested that higher serum selenium concentrations were associated with higher blood pressure levels and a higher prevalence of hypertension [95].

In summary, mere observational studies contain numerous confounding factors impeding interpretation and generalization. Recent data are scarce and do not provide a more definitive picture on whether selenium serum levels are a reliable biomarker in the development or monitoring of cardiovascular disease. Data on the role of selenium serum levels in cardiovascular disease remain inconclusive and in part contradictory. Transfer of knowledge from interventional supplementation studies to the role of selenium as a biomarker and *vice versa* is difficult.

Apart from selenium blood levels, direct measurement of selenoproteins as biomarkers in cardiovascular disease has also been investigated. Here, selenoprotein P has been proposed as a superior diagnostic marker of septic shock as compared to GPx [96]. Initial studies reported its role on atherosclerosis and coronary heart disease [97]. However, further trials in larger patient cohorts are needed to provide reliable data about the diagnostic use and utility of selenoprotein P and other biomarkers for the monitoring of cardiovascular disease (CVD) in clinical practice.

#### 3.3.2. Selenium in Myocardial Infarction and Cardiovascular Stress

In the acute phase following myocardial infarction and especially subsequent reperfusion, oxidative damage is paramount. One major source for myocardial ROS lies within the mitochondria and its production is linked to glutathione [98,99,100]. Of the 25 selenoproteins known today, GPx, thioredoxin reductases and methionine sulfoxide reductase B1 have especially been linked to cardiovascular stress [37]. A correlation between the extent of myocardial infarction as measured by peak Troponin I release and serum selenium levels has been shown in a small study on 55 patients with acute myocardial infarction [101]. The AtheroGene study showed no effect of low selenium concentrations on stable angina pectoris but found them to be associated with future cardiovascular death in patients with acute coronary syndrome [102].

#### 3.3.3. Prophylactic Selenium Supplementation

According to current knowledge derived from high-quality prospective studies, poor selenium nutritional status defined as true selenium deficiency or low serum/plasma selenium levels is considered a risk factor for CVD. However, data from trials on selenium supplementation are still inconclusive and have not shown major benefits [103]. So far, few randomized controlled trials (RCTs) have evaluated the effect of high-dose selenium intake on relevant cardiovascular endpoints. In a systematic review and meta-analysis of the literature, after aggregating 14 trials (*n* = 17,776 patients), Flores Mateo and co-workers [16] found a statistically significant inverse correlation between selenium levels and the incidence of atherosclerotic CHD. Furthermore, four RCTs supplementing antioxidant cocktails with selenium (daily dose range from 75 μg to 200 μg) and other trace elements and vitamins did not show any positive effect on clinical outcomes [16]. In 2014, another meta-analysis comprising 12 trials and over 19,000 participants [18] did not find any significant effect of selenium supplementation on overall mortality, mortality related to CVD and all fatal and non-fatal cardiovascular events. In addition, the authors found a significant reduction in non high-density lipoprotein (non-HDL) cholesterol levels. Nonetheless, the effects of selenium substitution on lipid profile remain partially understood [88]. In fact, data from the French multicenter trial *Supplementation with Antioxidant Vitamins and Minerals* (SU.VI.MAX) study [104,105] showed that selenium supplementation (100 μg day^−1^) was associated with higher triglyceride levels, and with lower HDL- cholesterol levels among men [88]. Moreover, women supplemented with selenium exhibited higher total cholesterol levels at the end of follow-up and men were more likely to use lipid-lowering drugs [88,104,105].

Unfortunately, no published RCTs are available that evaluated the efficacy of selenium supplementation in heart failure with the only exception of Keshan disease [106]. Previous studies demonstrated poor outcome in Chagasic cardiomyopathy, which was associated with low selenium levels in an animal model. Currently, the Selenium Treatment and Chagasic Cardiopathy (STCC) study [107], is evaluating in adult patients with mild or moderate global left ventricular systolic dysfunction, the efficacy of selenium supplementation given as a daily dose of 100 μg sodium selenite during 12 consecutive months compared to placebo.

According to current knowledge, there is insufficient evidence to support protective effects of selenium therapy in cardiovascular prevention. Therefore, large observational studies and well-powered high-quality RCTs are needed across populations from different geographical regions with different levels of selenium intake. Furthermore, trials results should be stratified by baseline plasma/serum selenium concentration of patients enrolled into randomized groups. Lastly, the composition of selenium should be considered cautiously, since it is of high relevance whether selenium is active in its inorganic form (selenite and redox derivatives) or needs to be organified to perform its biologic actions. Notwithstanding, we believe that before large scale and well-designed RCTs are developed, a phase II dose ranging clinical trial with prospective controls should be conducted in high-risk populations with the aim to determine the optimal and safe dose of chronic selenium intake as a prophylactic agent of CVD.

## 4. The Significance of Selenium in Cardiac Surgery

Worldwide, approximately one million patients per annum are in need of cardiac surgery. In the next decade, demand is expected to increase beyond this number due to an aging population [108]. Despite considerable advances in myocardial preservation strategies, cardiac surgery still remains associated with severe complications. The incidence will intensify in the future as cardiac surgery is increasingly being performed on an elderly population with increased numbers of simultaneous medical conditions and complex coronary lesions. Analyses of patient databases indicated that major adverse events including death, myocardial infarction, cardiac arrest and failure, renal failure, stroke, gastrointestinal complications and respiratory failure occur in up to 16% of all patients already after first admission to the hospital [109]. Three main pathophysiological mechanisms are associated with the majority of these systemic complications: Ischemia, reperfusion injury and perioperative inflammation [110,111,112].

**Figure 3:**
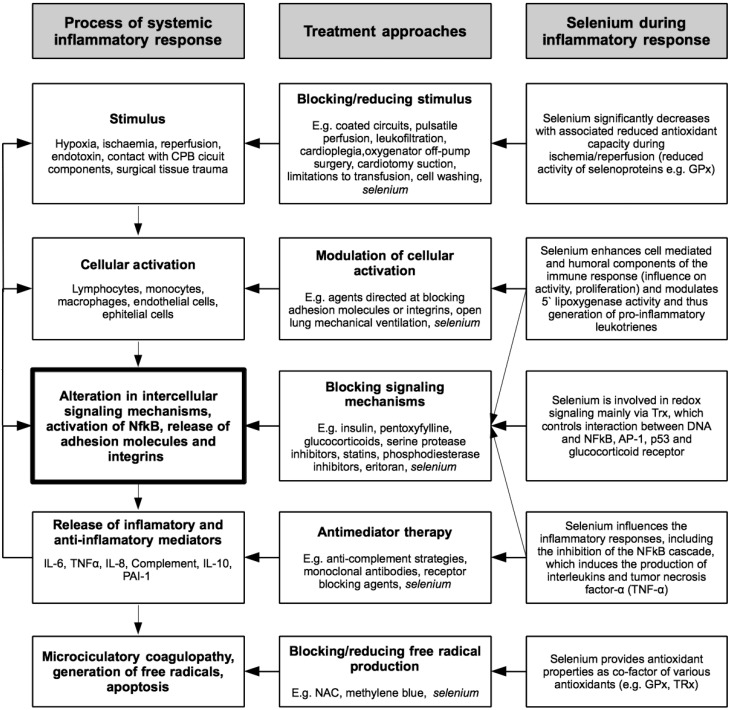
Systemic inflammatory response and treatment approaches in cardiac surgery (adapted from Hall [111]).

Patients undergoing on-pump cardiac surgery are subjected to various ischemic stimuli: (a) induction of cardioplegic arrest, (b) micro embolic events, (c) reperfusion of the myocardium by surgical revascularization and (d) termination of cardioplegic arrest [110,112]. Those factors activate a well-documented inflammatory response associated with manifestation of pyrexia, leukocytosis, tachycardia, hypotension, tissue fluid accumulation and organ failure [111]. This understanding of the causal mechanisms leads to various approaches utilized to decrease this stress response, reduce organ failure and improve patients’ outcomes after cardiac surgery. There are both pharmacological strategies, such as perioperative glucocorticoid administration, and non-pharmacological approaches, such as use of off-pump surgery, that address different aspects of this pathophysiological mechanism (Figure 3). In cardiac surgical patients, selenium is postulated to provide beneficial properties at each step in the cascade from stimulus to organ injury. Importantly, selenium and its antioxidant selenoenzyme GPx is capable of neutralizing both types of free radicals in the antioxidant intracellular system [81,112].

Therefore, the time course of perioperative selenium has been evaluated in a recent observational study. Interestingly, the majority of cardiac surgical patients exhibited a significant selenium deficiency already prior to surgery, which was further aggravated intraoperatively [113]. Moreover, the observed intraoperative lowering of circulating selenium levels was associated with the later development of postoperative multiorgan dysfunction. Koszta and colleagues confirmed these results and demonstrated the significance of low preoperative selenium levels on the development of postoperative organ dysfunctions [114]. These observations have stimulated several clinical trials in which the efficacy of selenium supplementation will be analyzed.

To provide an overview, we have summarized in Table 1 all clinical trials (completed and on-going) investigating the effects of selenium supplementation (mono- and combined therapies in patients scheduled for cardiac surgery.

**Table 1:** Overview of clinical trials investigating the role of selenium in patients undergoing cardiac surgery.

Ref.	Study Design	Population	Selenium Salt from and Dosing Regime	Outcomes
Leong 2010 [115]	Randomized controlled trial (double-blind)	Patients undergoing elective CABG and/or valve surgery *n* = 117	Coenzyme Q_10_, magnesium orotate, lipoic acid, omega-3 fatty acids and selenium *vs.* placebo (approximately 2 months before and 1 month after surgery)	Metabolic therapy reduced plasma troponin I, 24 h postoperatively from 1.5 (1.2–1.8) (geometric mean 95% CI) μg L L^−1^, to 2.1 (1.8–2.6) μg L^−1^ (*p* = 0.003) and shortened the mean length of postoperative hospital stay by 1.2 days from 8.1 (7.5–8.7) to 6.9 (6.4–7.4) days (*p* = 0.004) and reduced hospital costs. Metabolic therapy was inexpensive and had no clinically significant side effects.
Stoppe 2011 [113]	Prospective observational study	Patients scheduled for cardiac surgery with CPB *n* = 60	-	Fifty patients exhibited a significant selenium deficiency already before surgery. In all patients, blood levels of selenium, copper, and zinc were significantly reduced after end of surgery when compared to preoperative values (selenium: 89.05 ± 12.65 to 70.84 ± 10.46 µg; zinc: 5.15 ± 0.68 to 4.19 ± 0.73 mg L^−1^; copper: 0.86 ± 0.15 to 0.65 ± 0.14 mg L^−1^; *p* < 0.001). Selenium concentrations at end of surgery were independently associated with the postoperative occurrence of multiorgan failure (*p* = 0.0026, odds ratio 0.8479, 95% confidence interval 0.7617 to 0.9440).
Koszta 2012 [114]	Prospective observational study	Patients scheduled for cardiac surgery with CPB *n* = 197	-	Selenium levels were significantly lower in non-survivors 102.2 ± 19.5 μg L^−1^ compared with survivors 111.1 ± 16.9 μg L^−1^ (*p* = 0.047), and the mean age, European System for Cardiac Operative Risk Evaluation (EuroSCORE) values, and troponin concentrations were significantly higher in the non-survivors. Lower selenium levels identified as a risk factor for postoperative mortality.
Stoppe 2013 [116]	Prospective observational study	Patients scheduled for cardiac surgery with CPB *n* = 104	Intravenous bolus of 2.000 μg selenium after induction of anesthesia and 1.000 μg selenium every day further during ICU stay	Preoperative sodium-selenite administration increased selenium blood concentrations to normal values on ICU admission, but failed to prevent a significant decrease of circulating selenium on the first postoperative day.
Stevanovic 2014 [110]	Randomized controlled trial (comparison: Off- *vs.* on-pump CABG)	Patients undergoing elective CABG *n* = 40	-	Both groups showed a comparable decrease of circulating selenium concentrations. Likewise, levels of oxidative stress and IL-6 were comparable in both groups. Selenium levels correlated with antioxidant capacity (GPx: *r* = 0.720; *p* < 0.001) and showed a negative correlation to myocardial damage (CK-MB: *r* = −0.571, *p* < 0.001). Low postoperative selenium levels had a high predictive value for the occurrence of any postoperative complication.
Sustain CSX Trial 2014 [117]	Randomized controlled trial (double-blind)	Patients undergoing CABG plus valve surgery, multiple valve replacement surgery, patients with a high perioperative risk profile (≥5% EuroSCORE II). *n* = 1400	Intravenous bolus of 2.000 μg selenium after induction of anesthesia and 1.000 μg selenium every day further during ICU stay	On-going, recruiting
Haberthuer ClinicalTrials.gov Identifier: NCT01141556	Randomized controlled trial (double-blind)	Elective all-cause cardiac surgery *n* = 410	Loading dose of 4.000 μg, daily dosage of 1.000 μg of selenium *vs.* placebo	On-going, on analysis

CABG, coronary artery bypass graft; CPB, cardiopulmonary bypass; ICU, Intensive Care Unit; IL-6, Interleukin 6; GPx, Glutathione peroxidases; creatine kinase isoenzyme, CK-MB.

We have identified seven clinical studies investigating the role of selenium in patients undergoing cardiac surgery. Two trials are still on going. For one of these on-going trials, enrolment is completed but the results are still awaiting publication. Besides the previously discussed observational studies, one open-label trial evaluated the safety and efficacy of high-dose selenium supplementation in cardiac surgery patients. The investigators revealed a significantly reduced extent of organ injury at the 1st postoperative day in comparison to a historic control group [116]. Three double blind, randomized controlled studies evaluated the efficacy of selenium supplementation on patient outcome. While two studies are still on-going, the results of a randomized controlled trial performed by Leong and colleagues demonstrated a significantly reduced incidence of myocardial injury in patients that received a metabolic therapy together with a dose of selenium. However, the influence of additional antioxidants, other than selenium, cannot be ruled out in this study.

Given these limited data, results from large, well planned randomized controlled trials are still needed before any recommendations can be made on the pre-, peri- or postoperative dosage of selenium that might compensate for any pre-existing low selenium status and/or acquired selenium deficiency due to cardiac surgical interventions.

## 5. Future Directions and Therapeutic Implications

Emerging evidence suggests a key role of selenium in the setting of cardiac surgery, which thus expands its potential implications in the setting of acute myocardial ischemia/reperfusion. Previous studies have demonstrated that decreases in intraoperative selenium levels correlated with the duration of cardiopulmonary-bypass [113]. Therefore, selenium is presumed to be of high relevance especially in high-risk cardiac surgery patients with prolonged surgical procedures, resulting in a significantly increased duration of cardiopulmonary bypass (CPB) [117].

The technical progress in ventricular assist devices, combined with a decreasing rate of organ donors has resulted in an increasing use of this novel therapy, either as a bridge to transplantation or a destination therapy. Unfortunately, this promising approach to enhance survival and improve functional capacity is still associated with a notable number of infections, bleeding and thrombosis. Selenium’s beneficial effect on the immune function [9,10,11] and its well established antioxidant properties [4,118] could render this trace element an attractive complementary option in support of patients after ventricular device implantation. The continuous circulation of blood through the circuit results in a sustained inflammatory response with constant generation of reactive oxygen species [119]. Since selenium status is known to decrease with duration of CPB [113], it remains a challenge to clarify if selenium supplementation is capable of reducing the extent of inflammation and overall complication rates after device implantation.

As stated before, oxidative stress is known to significantly contribute to the development of myocardial damage and dysfunction. However, apart from measuring circulating selenium levels and the activity of selenium dependent enzymes, it is largely unknown whether the present genotype and polymorphism of patients might affect selenium distribution and subsequently the development of CVD. Furthermore, it remains speculative if the underlying genotype might influence the physiological response to additional selenium supplementation. Until today, the polymorphisms of the several single nucleotide repeats (SNP) in selenoprotein genes have been identified including Sel1, GPx1, GPx4, Sel15 SelP [120,121]. In particular, the latter significantly increased after selenium supplementation and the colorectal cancer risk in patients was reduced [120]. Since SelP represents the major transport form of selenium, accounting for approximately 60% selenium in plasma [122], the underlying genotype may have a significant impact on the development, progression and resolution of chronic and acute myocardial diseases. While emerging evidence indicates the significance of gene variations on the development of chronic and acute diseases, such as cancer or the chronic, osteoarthropathy Kashin Beck Disease [123], further studies are needed to characterize the functional role and clinical significance of SNPs in the context of CVD.

Emerging evidence indicates that micronutrients such as selenium may modulate disease risk and affect health outcomes via effects on the epigenome. Epigenetic effects encompass alterations of gene expression, whereas the primary DNA remains unaffected. These studies were driven by investigations, which speculated that a dietary exposure may have health consequences years or decades later. The underlying mechanisms are thought to be due to an effect of selenium on the three distinct and closely interacting mechanisms: Histone modification, DNA methylation and non-coding microRNAs, that regulate gene expression throughout the whole life course (reviewed in [124]). Therefore, these complex interactions and the responsiveness of epigenetic markers to selenium may open new therapeutic approaches to modulate the development and progression of CVD.

Given the existing evidence about the clinical relevance of variants in selenoprotein genes and the demonstrated epigenetic effects of selenium, one may suppose that these findings could serve as a future diagnostic tool for an individualized risk stratification in patients with cardiovascular diseases or in patients with planned cardiac surgery.

In extension to these therapeutic possibilities, there is experimental evidence that antioxidant treatment may ameliorate organ function after transplantation [125]. It would therefore be interesting to further elucidate whether patients with heart transplantation and exposure to an overwhelming inflammatory response in the period post-transplantation, might benefit from selenium supplementation.

## 6. Conclusions

In summary, there exists an impressive body of evidence about the several important functions of selenium and its selenoproteins in the cardiovascular system, which are mainly due to its well-known antioxidant characteristics. Although the role of selenium supplementation in the prevention of cardiovascular diseases is inconclusive, it is important to clarify if selenium deficiency leads to new health implications, particularly in relation to acute cardiovascular disease, where patients are exposed to myocardial I/R and increased oxidative stress.

Further clinical studies are needed to characterize the significance of selenium and selenoproteins in physio- and pathophysiological processes of the human body and to translate the existing knowledge from the laboratory bench to the bedside.

## References

[1] Muth O.H., Oldfield J.E., Remmert L.F., Schubert J.R. (1958). Effects of selenium and vitamin E on white muscle disease. Science.

[2] Navarro-Alarcon M., Cabrera-Vique C. (2008). Selenium in food and the human body: A review. Sci. Total Environ..

[3] Thomson C.D. (2004). Assessment of requirements for selenium and adequacy of selenium status: A review. Eur. J. Clin. Nutr..

[4] Fairweather-Tait S.J., Bao Y., Broadley M.R., Collings R., Ford D., Hesketh J.E., Hurst R. (2011). Selenium in human health and disease. Antioxid. Redox Signal..

[5] Yang G., Ge K., Chen J., Chen X. (1988). Selenium-related endemic diseases and the daily selenium requirement of humans. World Rev. Nutr. Diet..

[6] MacFarquhar J.K., Broussard D.L., Melstrom P., Hutchinson R., Wolkin A., Martin C., Burk R.F., Dunn J.R., Green A.L., Hammond R. (2010). Acute selenium toxicity associated with a dietary supplement. Arch. Intern. Med..

[7] Rayman M.P. (2008). Food-chain selenium and human health: Emphasis on intake. Br. J. Nutr..

[8] Lech T. (2002). Suicide by sodium tetraoxoselenate(VI) poisoning. Forensic Sci. Int..

[9] Arthur J.R., McKenzie R.C., Beckett G.J. (2003). Selenium in the immune system. J. Nutr..

[10] McKenzie R.C., Rafferty T.S., Beckett G.J. (1998). Selenium: An essential element for immune function. Immunol. Today.

[11] Kiremidjian-Schumacher L., Roy M. (1997). Selenium and immune function. Z. Ernahrungswiss..

[12] Ahrens I., Ellwanger C., Smith B.K., Bassler N., Chen Y.C., Neudorfer I., Ludwig A., Bode C., Peter K. (2008). Selenium supplementation induces metalloproteinase-dependent l-selectin shedding from monocytes. J. Leukoc. Biol..

[13] Heyland D.K., Dhaliwal R., Suchner U., Berger M.M. (2004). Antioxidant nutrients: A systematic review of trace elements and vitamins in the critically ill patient. Intensive Care Med..

[14] Forman H.J., Torres M. (2002). Reactive oxygen species and cell signaling: Respiratory burst in macrophage signaling. Am. J. Respir. Crit. Care Med..

[15] Arbogast S., Ferreiro A. (2010). Selenoproteins and protection against oxidative stress: Selenoprotein N as a novel player at the crossroads of redox signaling and calcium homeostasis. Antioxid. Redox Signal..

[16] Flores-Mateo G., Navas-Acien A., Pastor-Barriuso R., Guallar E. (2006). Selenium and coronary heart disease: A meta-analysis. Am. J. Clin. Nutr..

[17] Rees K., Hartley L., Day C., Flowers N., Clarke A., Stranges S. (2013). Selenium supplementation for the primary prevention of cardiovascular disease. Cochrane Database Syst. Rev..

[18] Loscalzo J. (2014). Keshan disease, selenium deficiency, and the selenoproteome. N. Engl. J. Med..

[19] Schrauzer G.N. (2000). Selenomethionine: A review of its nutritional significance, metabolism and toxicity. J. Nutr..

[20] Hardy G., Hardy I., Manzanares W. (2012). Selenium supplementation in the critically ill. Nutr. Clin. Pract..

[21] Reeves M.A., Hoffmann P.R. (2009). The human selenoproteome: Recent insights into functions and regulation. Cell. Mol. Life Sci..

[22] Grumolato L., Ghzili H., Montero-Hadjadje M., Gasman S., Lesage J., Tanguy Y., Galas L., Ait-Ali D., Leprince J., Guerineau N.C. (2008). Selenoprotein T is a PACAP-regulated gene involved in intracellular Ca^2+^ mobilization and neuroendocrine secretion. FASEB J..

[23] Prevost G., Arabo A., Jian L., Quelennec E., Cartier D., Hassan S., Falluel-Morel A., Tanguy Y., Gargani S., Lihrmann I. (2013). The PACAP-regulated gene selenoprotein T is abundantly expressed in mouse and human beta-cells and its targeted inactivation impairs glucose tolerance. Endocrinology.

[24] Papp L.V., Lu J., Holmgren A., Khanna K.K. (2007). From selenium to selenoproteins: Synthesis, identity, and their role in human health. Antioxid. Redox Signal..

[25] Huang Z., Rose A.H., Hoffmann P.R. (2012). The role of selenium in inflammation and immunity: From molecular mechanisms to therapeutic opportunities. Antioxid. Redox Signal..

[26] Brigelius-Flohé R., Maiorino M. (2013). Glutathione peroxidases. Biochim. Biophys. Acta.

[27] Flohe L., Gunzler W.A., Schock H.H. (1973). Glutathione peroxidase: A selenoenzyme. FEBS Lett..

[28] Rotruck J.T. (1973). Selenium: Biochemical role as a component of glutathione peroxidase. Science.

[29] Yoshida T., Watanabe M., Engelman D.T., Engelman R.M., Schley J.A., Maulik N., Ho Y.S., Oberley T.D., Das D.K. (1996). Transgenic mice overexpressing glutathione peroxidase are resistant to myocardial ischemia reperfusion injury. J. Mol. Cell. Cardiol..

[30] Yoshida T., Maulik N., Engelman R.M., Ho Y.S., Magnenat J.L., Rousou J.A., Flack J.E., Deaton D., Das D.K. (1997). Glutathione peroxidase knockout mice are susceptible to myocardial ischemia reperfusion injury. Circulation.

[31] Lim C.C., Bryan N.S., Jain M., Garcia-Saura M.F., Fernandez B.O., Sawyer D.B., Handy D.E., Loscalzo J., Feelisch M., Liao R. (2009). Glutathione peroxidase deficiency exacerbates ischemia-reperfusion injury in male but not female myocardium: Insights into antioxidant compensatory mechanisms. Am. J. Physiol. Heart Circ. Physiol..

[32] Jin R.C., Mahoney C.E., Anderson L.C., Ottaviano F., Croce K., Leopold J.A., Zhang Y.Y., Tang S.S., Handy D.E., Loscalzo J. (2011). Glutathione peroxidase-3 deficiency promotes platelet-dependent thrombosis *in vivo*. Circulation.

[33] Brigelius-Flohé R., Banning A., Schnurr K. (2003). Selenium-dependent enzymes in endothelial cell function. Antioxid. Redox Signal..

[34] Hoffmann F.W., Hashimoto A.S., Lee B.C., Rose A.H., Shohet R.V., Hoffmann P.R. (2011). Specific antioxidant selenoproteins are induced in the heart during hypertrophy. Arch. Biochem. Biophys..

[35] Wortmann M., Schneider M., Pircher J., Hellfritsch J., Aichler M., Vegi N., Kölle P., Kuhlencordt P., Walch A., Pohl U. (2013). Combined deficiency in glutathione peroxidase 4 and vitamin E causes multiorgan thrombus formation and early death in mice. Circ. Res..

[36] Hollander J.M., Lin K.M., Scott B.T., Dillmann W.H. (2003). Overexpression of PHGPx and HSP60/10 protects against ischemia/reoxygenation injury. Free Radic. Biol. Med..

[37] Rose A.H., Hoffmann P.R. (2015). Selenoproteins and cardiovascular stress. Thromb. Haemost..

[38] Arner E.S., Holmgren A. (2000). Physiological functions of thioredoxin and thioredoxin reductase. Eur. J. Biochem. FEBS.

[39] Maulik N., Das D.K. (2008). Emerging potential of thioredoxin and thioredoxin interacting proteins in various disease conditions. Biochim. Biophys. Acta.

[40] Biterova E.I., Turanov A.A., Gladyshev V.N., Barycki J.J. (2005). Crystal structures of oxidized and reduced mitochondrial thioredoxin reductase provide molecular details of the reaction mechanism. Proc. Natl. Acad. Sci. USA.

[41] Holmgren A., Lu J. (2010). Thioredoxin and thioredoxin reductase: Current research with special reference to human disease. Biochem. Biophys. Res. Commun..

[42] Tamura T., Stadtman T.C. (1996). A new selenoprotein from human lung adenocarcinoma cells: Purification, properties, and thioredoxin reductase activity. Proc. Natl. Acad. Sci. USA.

[43] Lee S.R., Kim J.R., Kwon K.S., Yoon H.W., Levine R.L., Ginsburg A., Rhee S.G. (1999). Molecular cloning and characterization of a mitochondrial selenocysteine-containing thioredoxin reductase from rat liver. J. Biol. Chem..

[44] Sun Q.A., Kirnarsky L., Sherman S., Gladyshev V.N. (2001). Selenoprotein oxidoreductase with specificity for thioredoxin and glutathione systems. Proc. Natl. Acad. Sci. USA.

[45] Lundstrom J., Holmgren A. (1990). Protein disulfide-isomerase is a substrate for thioredoxin reductase and has thioredoxin-like activity. J. Biol. Chem..

[46] Lundstrom-Ljung J., Birnbach U., Rupp K., Soling H.D., Holmgren A. (1995). Two resident ER-proteins, CaBP1 and CaBP2, with thioredoxin domains, are substrates for thioredoxin reductase: Comparison with protein disulfide isomerase. FEBS Lett..

[47] Bjornstedt M., Hamberg M., Kumar S., Xue J., Holmgren A. (1995). Human thioredoxin reductase directly reduces lipid hydroperoxides by NADPH and selenocystine strongly stimulates the reaction via catalytically generated selenols. J. Biol. Chem..

[48] Bjornstedt M., Kumar S., Holmgren A. (1992). Selenodiglutathione is a highly efficient oxidant of reduced thioredoxin and a substrate for mammalian thioredoxin reductase. J. Biol. Chem..

[49] Gromer S., Gross J.H. (2002). Methylseleninate is a substrate rather than an inhibitor of mammalian thioredoxin reductase. Implications for the antitumor effects of selenium. J. Biol. Chem..

[50] Kumar S., Bjornstedt M., Holmgren A. (1992). Selenite is a substrate for calf thymus thioredoxin reductase and thioredoxin and elicits a large non-stoichiometric oxidation of NADPH in the presence of oxygen. Eur. J. Biochem. FEBS.

[51] Ganther H.E. (1999). Selenium metabolism, selenoproteins and mechanisms of cancer prevention: Complexities with thioredoxin reductase. Carcinogenesis.

[52] Tanaka K., Honda M., Takabatake T. (2001). Redox regulation of MAPK pathways and cardiac hypertrophy in adult rat cardiac myocyte. J. Am. Coll. Cardiol..

[53] Berndt C., Lillig C.H., Holmgren A. (2007). Thiol-based mechanisms of the thioredoxin and glutaredoxin systems: Implications for diseases in the cardiovascular system. Am. J. Physiol. Heart Circ. Physiol..

[54] Yamamoto M., Yang G., Hong C., Liu J., Holle E., Yu X., Wagner T., Vatner S.F., Sadoshima J. (2003). Inhibition of endogenous thioredoxin in the heart increases oxidative stress and cardiac hypertrophy. J. Clin. Investig..

[55] Ago T., Sadoshima J. (2006). Thioredoxin and ventricular remodeling. J. Mol. Cell. Cardiol..

[56] Wang L., Proud C.G. (2002). Ras/Erk signaling is essential for activation of protein synthesis by Gq protein-coupled receptor agonists in adult cardiomyocytes. Circ. Res..

[57] Schomburg L., Köhrle J. (2008). On the importance of selenium and iodine metabolism for thyroid hormone biosynthesis and human health. Mol. Nutr. Food Res..

[58] Mittag J., Behrends T., Hoefig C.S., Vennström B., Schomburg L. (2010). Thyroid hormones regulate selenoprotein expression and selenium status in mice. PLoS ONE.

[59] Bianco A.C., Salvatore D., Gereben B., Berry M.J., Larsen P.R. (2002). Biochemistry, cellular and molecular biology, and physiological roles of the iodothyronine selenodeiodinases. Endocr. Rev..

[60] Klein I., Ojamaa K. (2001). Thyroid hormone and the cardiovascular system. N. Engl. J. Med..

[61] Trivieri M.G., Oudit G.Y., Sah R., Kerfant B.G., Sun H., Gramolini A.O., Pan Y., Wickenden A.D., Croteau W., de Escobar G.M. (2006). Cardiac-specific elevations in thyroid hormone enhance contractility and prevent pressure overload-induced cardiac dysfunction. Proc. Natl. Acad. Sci. USA.

[62] Stadtman E.R. (2006). Protein oxidation and aging. Free Radic. Res..

[63] Moskovitz J., Bar-Noy S., Williams W.M., Requena J., Berlett B.S., Stadtman E.R. (2001). Methionine sulfoxide reductase (MsrA) is a regulator of antioxidant defense and lifespan in mammals. Proc. Natl. Acad. Sci. USA.

[64] Moskovitz J., Flescher E., Berlett B.S., Azare J., Poston J.M., Stadtman E.R. (1998). Overexpression of peptide-methionine sulfoxide reductase in Saccharomyces cerevisiae and human T cells provides them with high resistance to oxidative stress. Proc. Natl. Acad. Sci. USA.

[65] Kim H.Y., Gladyshev V.N. (2004). Methionine sulfoxide reduction in mammals: Characterization of methionine-R-sulfoxide reductases. Mol. Biol. Cell.

[66] Picot C.R., Perichon M., Lundberg K.C., Friguet B., Szweda L.I., Petropoulos I. (2006). Alterations in mitochondrial and cytosolic methionine sulfoxide reductase activity during cardiac ischemia and reperfusion. Exp. Gerontol..

[67] Lee B.C., Peterfi Z., Hoffmann F.W., Moore R.E., Kaya A., Avanesov A., Tarrago L., Zhou Y., Weerapana E., Fomenko D.E. (2013). MsrB1 and MICALs regulate actin assembly and macrophage function via reversible stereoselective methionine oxidation. Mol. Cell.

[68] Lu C., Qiu F., Zhou H., Peng Y., Hao W., Xu J., Yuan J., Wang S., Qiang B., Xu C. (2006). Identification and characterization of selenoprotein K: An antioxidant in cardiomyocytes. FEBS Lett..

[69] Meiler S., Baumer Y., Huang Z., Hoffmann F.W., Fredericks G.J., Rose A.H., Norton R.L., Hoffmann P.R., Boisvert W.A. (2013). Selenoprotein K is required for palmitoylation of CD36 in macrophages: Implications in foam cell formation and atherogenesis. J. Leukoc. Biol..

[70] Cox A.J., Lehtinen A.B., Xu J., Langefeld C.D., Freedman B.I., Carr J.J., Bowden D.W. (2013). Polymorphisms in the Selenoprotein S gene and subclinical cardiovascular disease in the Diabetes Heart Study. Acta Diabetol..

[71] Gao Y., Feng H.C., Walder K., Bolton K., Sunderland T., Bishara N., Quick M., Kantham L., Collier G.R. (2004). Regulation of the selenoprotein SelS by glucose deprivation and endoplasmic reticulum stress—SelS is a novel glucose-regulated protein. FEBS Lett..

[72] Xu G.L., Wang S.C., Gu B.Q., Yang Y.X., Song H.B., Xue W.L., Liang W.S., Zhang P.Y. (1997). Further investigation on the role of selenium deficiency in the aetiology and pathogenesis of Keshan disease. Biomed. Environ. Sci..

[73] Beck M.A., Levander O.A., Handy J. (2003). Selenium deficiency and viral infection. J. Nutr..

[74] Beck M.A., Handy J., Levander O.A. (2004). Host nutritional status: The neglected virulence factor. Trends Microbiol..

[75] Jun E.J., Ye J.S., Hwang I.S., Kim Y.K., Lee H. (2011). Selenium deficiency contributes to the chronic myocarditis in coxsackievirus-infected mice. Acta Virol..

[76] Beck M.A., Esworthy R.S., Ho Y.S., Chu F.F. (1998). Glutathione peroxidase protects mice from viral-induced myocarditis. FASEB J..

[77] Venardos K. (2004). Effects of dietary selenium on glutathione peroxidase and thioredoxin reductase activity and recovery from cardiac ischemia-reperfusion. J. Trace Elem. Med. Biol..

[78] Toufektsian M.C., Boucher F., Pucheu S., Tanguy S., Ribuot C., Sanou D., Tresallet N., de Leiris J. (2000). Effects of selenium deficiency on the response of cardiac tissue to ischemia and reperfusion. Toxicology.

[79] Tanguy S., Toufektsian M.C., Besse S., Ducros V., de Leiris J., Boucher F. (2003). Dietary selenium intake affects cardiac susceptibility to ischaemia/reperfusion in male senescent rats. Age Ageing.

[80] Pucheu S., Coudray C., Tresallet N., Favier A., de Leiris J. (1995). Effect of dietary antioxidant trace element supply on cardiac tolerance to ischemia-reperfusion in the rat. J. Mol. Cell. Cardiol..

[81] Venardos K.M., Perkins A., Headrick J., Kaye D.M. (2007). Myocardial ischemia-reperfusion injury, antioxidant enzyme systems, and selenium: A review. Curr. Med. Chem..

[82] Tanguy S., Boucher F., Besse S., Ducros V., Favier A., de Leiris J. (1998). Trace elements and cardioprotection: Increasing endogenous glutathione peroxidase activity by oral selenium supplementation in rats limits reperfusion-induced arrhythmias. J. Trace Elem. Med. Biol..

[83] Tanguy S., Morel S., Berthonneche C., Toufektsian M.C., de Lorgeril M., Ducros V., Tosaki A., de Leiris J., Boucher F. (2004). Preischemic selenium status as a major determinant of myocardial infarct size *in vivo* in rats. Antioxid. Redox Signal..

[84] Tanguy S., Rakotovao A., Jouan M.G., Ghezzi C., de Leiris J., Boucher F. (2011). Dietary selenium intake influences Cx43 dephosphorylation, TNF-α expression and cardiac remodeling after reperfused infarction. Mol. Nutr. Food Res..

[85] Rakotovao A., Tanguy S., Toufektsian M.C., Berthonneche C., Ducros V., Tosaki A., de Leiris J., Boucher F. (2005). Selenium status as determinant of connexin-43 dephosphorylation in *ex vivo* ischemic/reperfused rat myocardium. J. Trace Elem. Med. Biol..

[86] Rotruck J.T. (1972). Prevention of oxidative damage to rat erythrocytes by dietary selenium. J. Nutr..

[87] Aviram M., Fuhrman B. (1998). LDL oxidation by arterial wall macrophages depends on the oxidative status in the lipoprotein and in the cells: Role of prooxidants *vs.* antioxidants. Mol. Cell. Biochem..

[88] Navas-Acien A., Bleys J., Guallar E. (2008). Selenium intake and cardiovascular risk: What is new?. Curr. Opin. Lipidol..

[89] Xun P., Liu K., Morris J.S., Daviglus M.L., He K. (2010). Longitudinal association between toenail selenium levels and measures of subclinical atherosclerosis: The CARDIA trace element study. Atherosclerosis.

[90] Loscalzo J. (2014). Effects of nationwide addition of selenium to fertilizers on foods, and animal and human health in Finland: From deficiency to optimal selenium status of the population. J. Trace Elem. Med. Biol..

[91] Bleys J., Navas-Acien A., Guallar E. (2008). Serum selenium levels and all-cause, cancer, and cardiovascular mortality among US adults. Arch. Intern. Med..

[92] Joseph J. (2013). Selenium and cardiometabolic health: Inconclusive yet intriguing evidence. Am. J. Med. Sci..

[93] González J., Valls N., Brito R., Rodrigo R. (2014). Essential hypertension and oxidative stress: New insights. World J. Cardiol..

[94] Nawrot T.S., Staessen J.A., Roels H.A., Den Hond E., Thijs L., Fagard R.H., Dominiczak A.F., Struijker-Boudier H.A. (2007). Blood pressure and blood selenium: A cross-sectional and longitudinal population study. Eur. Heart J..

[95] Berthold H.K., Michalke B., Krone W., Guallar E., Gouni-Berthold I. (2012). Influence of serum selenium concentrations on hypertension: The Lipid Analytic Cologne cross-sectional study. J. Hypertens..

[96] Forceville X., Mostert V., Pierantoni A., Vitoux D., le Toumelin P., Plouvier E., Dehoux M., Thuillier F., Combes A., Selenoprotein P. (2009). Rather than Glutathione Peroxidase, as a Potential Marker of Septic Shock and Related Syndromes. Eur. Surg. Res..

[97] Yang S.J., Hwang S.Y., Choi H.Y., Yoo H.J., Seo J.A., Kim S.G., Kim N.H., Baik S.H., Choi D.S., Choi K.M. (2011). Serum selenoprotein P levels in patients with type 2 diabetes and prediabetes: Implications for insulin resistance, inflammation, and atherosclerosis. J. Clin. Endocrinol. Metab..

[98] Chen Y.R., Zweier J.L. (2014). Cardiac mitochondria and reactive oxygen species generation. Circ. Res..

[99] Grieve D.J., Byrne J.A., Cave A.C., Shah A.M. (2004). Role of oxidative stress in cardiac remodelling after myocardial infarction. Heart Lung Circ..

[100] Tsutsui H., Kinugawa S., Matsushima S. (2009). Mitochondrial oxidative stress and dysfunction in myocardial remodelling. Cardiovasc. Res..

[101] Kutil B., Ostadal P., Vejvoda J., Kukacka J., Cepova J., Alan D., Krüger A., Vondrakova D. (2010). Alterations in serum selenium levels and their relation to troponin I in acute myocardial infarction. Mol. Cell. Biochem..

[102] Lubos E., Sinning C.R., Schnabel R.B., Wild P.S., Zeller T., Rupprecht H.J., Bickel C., Lackner K.J., Peetz D., Loscalzo J. (2010). Serum selenium and prognosis in cardiovascular disease: Results from the AtheroGene study. Atherosclerosis.

[103] Eaton C.B., Abdul Baki A.R., Waring M.E., Roberts M.B., Lu B. (2010). The association of low selenium and renal insufficiency with coronary heart disease and all-cause mortality: NHANES III follow-up study. Atherosclerosis.

[104] Hercberg S., Galan P., Preziosi P., Bertrais S., Mennen L., Malvy D., Roussel A.M., Favier A., Briancon S. (2004). The SU.VI.MAX Study: A randomized, placebo-controlled trial of the health effects of antioxidant vitamins and minerals. Arch. Intern. Med..

[105] Hercberg S., Bertrais S., Czernichow S., Noisette N., Galan P., Jaouen A., Tichet J., Briancon S., Favier A., Mennen L. (2005). Alterations of the lipid profile after 7.5 years of low-dose antioxidant supplementation in the SU.VI.MAX Study. Lipids.

[106] McKeag N.A., McKinley M.C., Woodside J.V., Harbinson M.T., McKeown P.P. (2012). The role of micronutrients in heart failure. J. Acad. Nutr. Diet..

[107] Do Brasil P.E.A.A., de Souza A.P., Hasslocher-Moreno A.M., Xavier S.S., Passos S.R.L., Moreira M.D.F.R., de Oliveira M.S., da Silva G.M.S., Saraiva R.M., de Aguiar C.C.S. (2014). Selenium Treatment and Chagasic Cardiopathy (STCC): Study protocol for a double-blind randomized controlled trial. Trials.

[108] Grover A., Gorman K., Dall T.M., Jonas R., Lytle B., Shemin R., Wood D., Kron I. (2009). Shortage of cardiothoracic surgeons is likely by 2020. Circulation.

[109] Ghosh P., Schistek R., Unger F. (2004). Coronary revascularization in DACH: 1991–2002. Thorac. Cardiovasc. Surg..

[110] Stevanovic A., Coburn M., Menon A., Rossaint R., Heyland D., Schalte G., Werker T., Wonisch W., Kiehntopf M., Goetzenich A. (2014). The importance of intraoperative selenium blood levels on organ dysfunction in patients undergoing off-pump cardiac surgery: A randomised controlled trial. PLoS ONE.

[111] Hall R. (2013). Identification of inflammatory mediators and their modulation by strategies for the management of the systemic inflammatory response during cardiac surgery. J. Cardiothorac. Vasc. Anesth..

[112] Manzanares W., Langlois P.L., Heyland D.K. (2015). Pharmaconutrition With Selenium in Critically Ill Patients: What Do We Know?. Nutr. Clin. Pract..

[113] Stoppe C., Schalte G., Rossaint R., Coburn M., Graf B., Spillner J., Marx G., Rex S. (2011). The intraoperative decrease of selenium is associated with the postoperative development of multiorgan dysfunction in cardiac surgical patients. Crit. Care Med..

[114] Koszta G., Kacska Z., Szatmari K., Szerafin T., Fulesdi B. (2012). Lower whole blood selenium level is associated with higher operative risk and mortality following cardiac surgery. J. Anesth..

[115] Leong J.Y., van der Merwe J., Pepe S., Bailey M., Perkins A., Lymbury R., Esmore D., Marasco S., Rosenfeldt F. (2010). Perioperative metabolic therapy improves redox status and outcomes in cardiac surgery patients: A randomised trial. Heart Lung Circ..

[116] Stoppe C., Spillner J., Rossaint R., Coburn M., Schalte G., Wildenhues A., Marx G., Rex S. (2013). Selenium blood concentrations in patients undergoing elective cardiac surgery and receiving perioperative sodium selenite. Nutrition.

[117] Stoppe C., McDonald B., Rex S., Manzanares W., Whitlock R., Fremes S., Fowler R., Lamarche Y., Meybohm P., Haberthur C. (2014). SodiUm SeleniTe Adminstration IN Cardiac Surgery (SUSTAIN CSX-trial): Study design of an international multicenter randomized double-blinded controlled trial of high dose sodium-selenite administration in high-risk cardiac surgical patients. Trials.

[118] Robinson M.F., Godfrey P.J., Thomson C.D., Rea H.M., van Rij A.M. (1979). Blood selenium and glutathione peroxidase activity in normal subjects and in surgical patients with and without cancer in New Zealand. Am. J. Clin. Nutr..

[119] Laffey J.G., Boylan J.F., Cheng D.C.H. (2002). The systemic inflammatory response to cardiac surgery: Implications for the anesthesiologist. Anesthesiology.

[120] Méplan C., Nicol F., Burtle B.T., Crosley L.K., Arthur J.R., Mathers J.C., Hesketh J.E. (2009). Relative abundance of selenoprotein P isoforms in human plasma depends on genotype, se intake, and cancer status. Antioxid. Redox Signal..

[121] Méplan C., Hughes D.J., Pardini B., Naccarati A., Soucek P., Vodickova L., Hlavatá I., Vrána D., Vodicka P., Hesketh J.E. (2010). Genetic variants in selenoprotein genes increase risk of colorectal cancer. Carcinogenesis.

[122] Hill K.E., Xia Y., Akesson B., Boeglin M.E., Burk R.F. (1996). Selenoprotein P concentration in plasma is an index of selenium status in selenium-deficient and selenium-supplemented Chinese subjects. J. Nutr..

[123] Wang W.Z., Guo X., Duan C., Ma W.J., Zhang Y.G., Xu P., Gao Z.Q., Wang Z.F., Yan H., Zhang Y.F. (2009). Comparative analysis of gene expression profiles between the normal human cartilage and the one with endemic osteoarthritis. Osteoarthr. Cartil..

[124] Speckmann B., Grune T. (2015). Epigenetic effects of selenium and their implications for health. Epigenetics.

[125] Treska V., Kuntscher V., Hasman D., Neprasová P., Kobr J., Racek J., Trefil L., Hes O. (2002). Importance of selenium for the influence of ischemia-reperfusion syndrome after kidney transplantation from a non-heart beating donor in a pig model. Transplant. Proc..

